# Acute effects of high-dose corticosteroids on serum biomarker profiles in primary CNS demyelinating disease: A pilot study

**DOI:** 10.1177/20552173261487037

**Published:** 2026-09-07

**Authors:** Anastasia P Chumakova, Michael Demetriou, Michael Sy

**Affiliations:** 1 Department of Neurology, 8788 University of California, Irvine, CA, USA

**Keywords:** Multiple sclerosis, relapsing-remitting, neuromyelitis optica, myelin-oligodendrocyte glycoprotein, methylprednisolone

## Abstract

**Background:**

Acute inflammatory relapses in primary CNS demyelinating diseases, including relapsing multiple sclerosis (RMS), MOG-antibody-associated disease (MOGAD), and neuromyelitis optica spectrum disorder (NMOSD), drive hospitalization, disability, and socioeconomic burden. Blood-based biomarkers are increasingly used for relapse detection and treatment monitoring.

**Objective:**

Investigate effects of corticosteroids on short-term dynamics of serum biomarkers.

**Methods:**

We conducted a pilot prospective study of 22 patients with acute neuro-inflammatory conditions, assessing changes in an 18 protein serum biomarker panel (Octave MSDA) and composite disease scores before and after high-dose intravenous methylprednisolone (IVMP; 1000 mg/day for 3 days). Samples were collected within 24 h before and after treatment.

**Results:**

In 14 RMS patients, IVMP produced significant decreases in GFAP, Serpin A9, FLRT2, contactin-2, protogenin, MOG, CXCL9, CD6, BAFF (TNFSF13B), IL-12B, and CDCP1. Composite MSDA scores showed paradoxical post-IVMP increases in overall disease activity, neuroinflammation, immunomodulation, and neuroaxonal integrity, with decreased myelin biology scores. In a pooled non-RMS group (MOGAD, NMOSD, idiopathic optic neuritis, and transverse myelitis), CNTN2 and IL-12B exhibited similar corticosteroid responsive reductions.

**Conclusions:**

These findings suggest that high-dose corticosteroids rapidly and significantly alter multiple serum biomarkers and composite activity scores across demyelinating conditions, underscoring implications for biomarker timing, interpretation, and future validation.

## Introduction

Acute inflammatory relapses are a central feature of relapsing multiple sclerosis (RMS) and related primary demyelinating disorders such as MOG-antibody-associated disease (MOGAD) and neuromyelitis optica spectrum disorder (NMOSD). These attacks frequently necessitate hospitalization, can result in stepwise accumulation of neurological disability, and carry substantial socioeconomic consequences. Reliable tools for quantifying inflammatory activity in the acute setting are therefore of considerable clinical interest.

Blood-derived biomarkers are emerging as a practical complement to imaging and clinical assessment for both relapse detection and longitudinal monitoring of disease-modifying therapies (DMTs).^1^ The Octave Biosciences Multiple Sclerosis Disease Activity panel (Octave MSDA panel) is a clinically available multianalyte assay validated in 614 cross-sectional serum samples, and includes 18 serum proteins that were associated with clinical relapse within 30 days and MRI gadolinium enhancement within 60 days of sample collection. It integrates analyte values into composite scores reflecting overall disease activity, neuroinflammation, immunomodulation, neuroaxonal integrity, and myelin biology. The Disease Activity score provides validated, clinically meaningful levels (high, moderate, and low).^2^ Several of the biomarkers included in the Octave MSDA panel have been reported to change with corticosteroid exposure, but the short-term dynamics of the full panel and effect on the composite scores during steroid treatments have not been characterized.

High-dose intravenous methylprednisolone (IVMP) is standard first-line therapy for acute demyelinating relapses across RMS, MOGAD, and NMOSD. Because biomarker testing is often used during a relapse to help aid in disease monitoring, understanding how corticosteroids alter individual protein concentrations and derived scores over a time frame of days is essential for accurate interpretation. Furthermore, it is unknown whether steroid-induced biomarker changes are similar across RMS and non-RMS demyelinating disorders.

In this pilot study, we aimed to (1) quantify short-term changes in individual proteins and composite MSDA scores following a standard 3-day course of high-dose IVMP in RMS; and (2) explore whether analogous corticosteroid-responsive patterns are observed in non-RMS demyelinating conditions.

## Methods

### Study design and patient selection

We conducted a single-center prospective pilot study of patients hospitalized with acute inflammatory relapses of primary CNS demyelinating disease. Eligible patients had a clinical syndrome consistent with acute neuro-inflammatory relapse.

Key inclusion features for the acute relapse cohort were:

- New or worsening neurological symptoms with symptom evolution consistent an inflammatory demyelinating event;
- Clinical decision to treat with high-dose IVMP (1000 mg/day) for at least 3 days.

We recruited 91 total patients with 22 patients included in this study. Patients with sample collections outside of the intended time frame, alterations in their treatment regimen, and patients with final diagnosis other than neuro-inflammatory conditions were excluded from further analysis.

### Treatment and sampling

All patients received high-dose IVMP 1000 mg daily for a planned 3-day course. Serum samples were collected at two time points:

- Pre-treatment - before initiation of IVMP. Mean time between collection and first infusion of IVMP was 8.66 h (median = 6.05 h, SD = 10.5 h).
- Post-treatment - after completion of the third IVMP dose. Mean time between completion of the last infusion and sample collection was 0.5 h (median = 1.8 h, SD = 11.3 h).

Clinical data including demographic characteristics, final diagnosis, laboratory and imaging findings, need for DMT escalation and outcome measures were collected from the medical record. All patients underwent evaluation with MRI during the hospitalization associated with treatment. MRI evidence of new inflammatory lesions and gadolinium-enhancing lesions was collected based on clinically indicated imaging modalities.

### Biomarker assessment

Serum samples were analyzed using the Octave MSDA panel, which quantifies 18 serum proteins and generates multiple composite scores (overall disease activity, neuroinflammation, immunomodulation, neuroaxonal integrity, and myelin biology).

### Statistical analysis

Data processing, statistical analyses, and visualization were performed using R software (version 4.2.0) with the following packages: ggpubr, ggplot2, dplyr, forcats, hrbrthemes, tibble, janitor, lubridate, tidyverse, finalfit, gridExtra, reshape, chron, rstatix, emmeans.

For longitudinal analysis, we evaluated paired pre and post-IVMP samples by paired *T* test (for continuous variables) or by paired Wilcox rank sum test (for rank variables) with FDR (Benjamini-Hochberg) correction for multiple comparisons.

## Results

**Figure 1.**
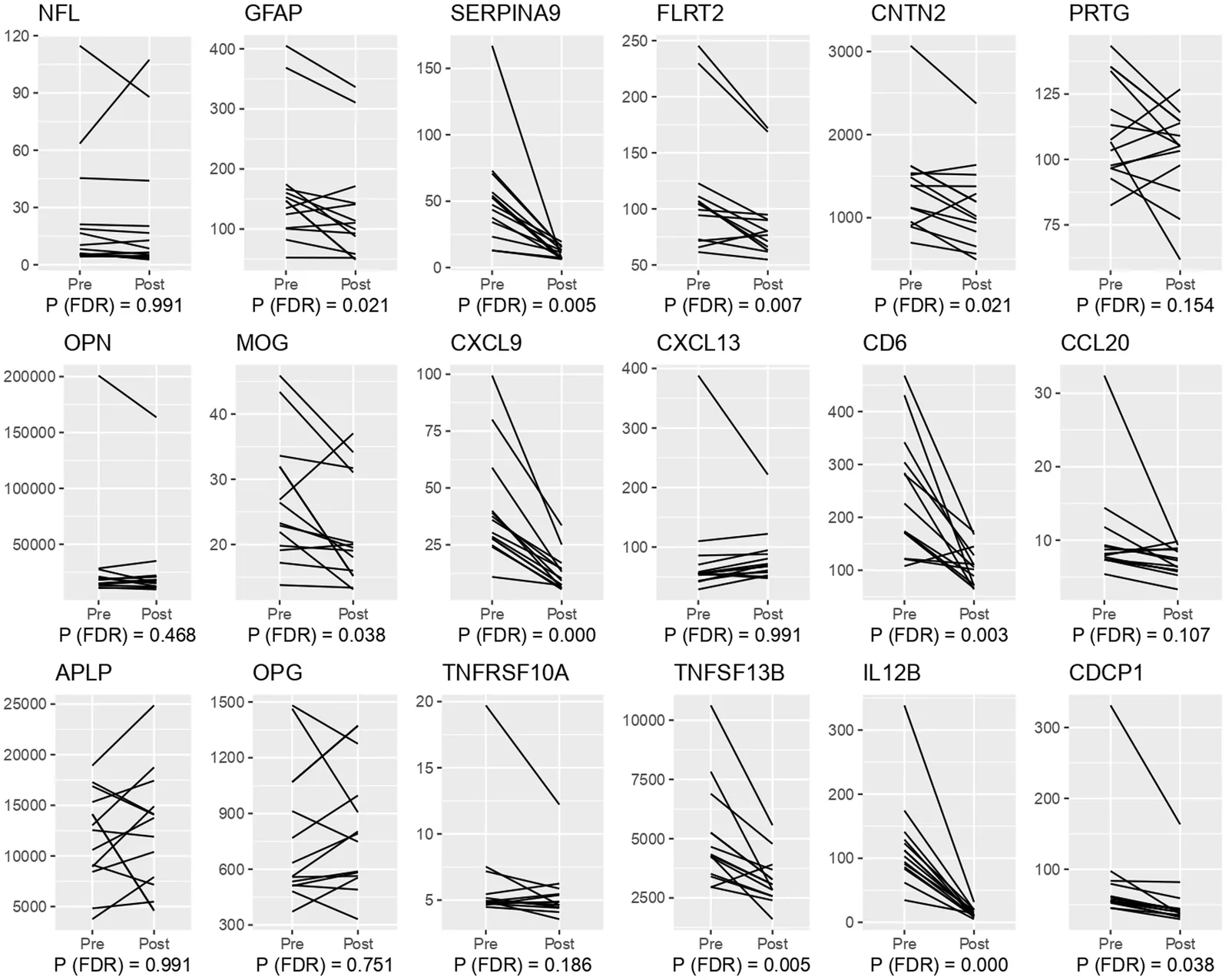
High-dose corticosteroids cause significant change in multiple serum biomarkers in patients with RMS. Absolute serum concentrations are presented. *P*-value is derived from paired *T*-test comparisons with FDR multiple comparisons correction.

We examined dynamic changes in the 14 RMS patients for whom paired pre and post-IVMP samples were available. Mean time from symptoms onset to first sample collection was 16 days (median = 8.6 days, SD = 17 days) (Table 1). Four patients had no enhancing lesions identified on MRI. High-dose IVMP was associated with statistically significant decreases in multiple serum proteins. Specifically, we observed significant reductions in: GFAP, Serpin A9, FLRT2, Contactin-2, MOG, CXCL9, CD6, BAFF (TNFSF13B), Interleukin-12B, CDCP1 (Figure 1). We saw similar changes in serum biomarkers when evaluating a subgroup of 10 RMS patients with MRI evidence of disease activity in post-hoc analysis (Supplemental Figure 1).

**Table 1.** Study demographics.

		MS	MOGAD	NMOSD	TM	ULON	*P*-value
Age	Mean (SD)	36.0 (13.0)	47.8 (15.7)	32.7 (NA)	39.4 (NA)	61.5 (19.0)	.165
Sex	Female	6 (42.9)	2 (50.0)	1 (100.0)		2 (100.0)	.369
	Male	8 (57.1)	2 (50.0)		1 (100.0)		
Days between symptom onset and first blood collection	Mean (SD)	18.8 (20.8)	11.3 (8.1)	14.3 (NA)	6.2 (NA)	11.7 (7.9)	.913
Gad+ lesion localization	None	3 (21.4)					.036
	Optic nerve	2 (14.3)	4 (100.0)			2 (100.0)	
	Brain	4 (28.6)					
	Spine	4 (28.6)			1 (100.0)		
	Multiple sites	1 (7.1)		1 (100.0)			

MS: multiple sclerosis; NMOSD: neuromyelitis optica spectrum disorder; MOGAD: myelin oligodendrocyte-glycoprotein associated disease; TM: idiopathic transverse myelitis; ULON: unilateral idiopathic optic neuritis.

**Figure 2.**
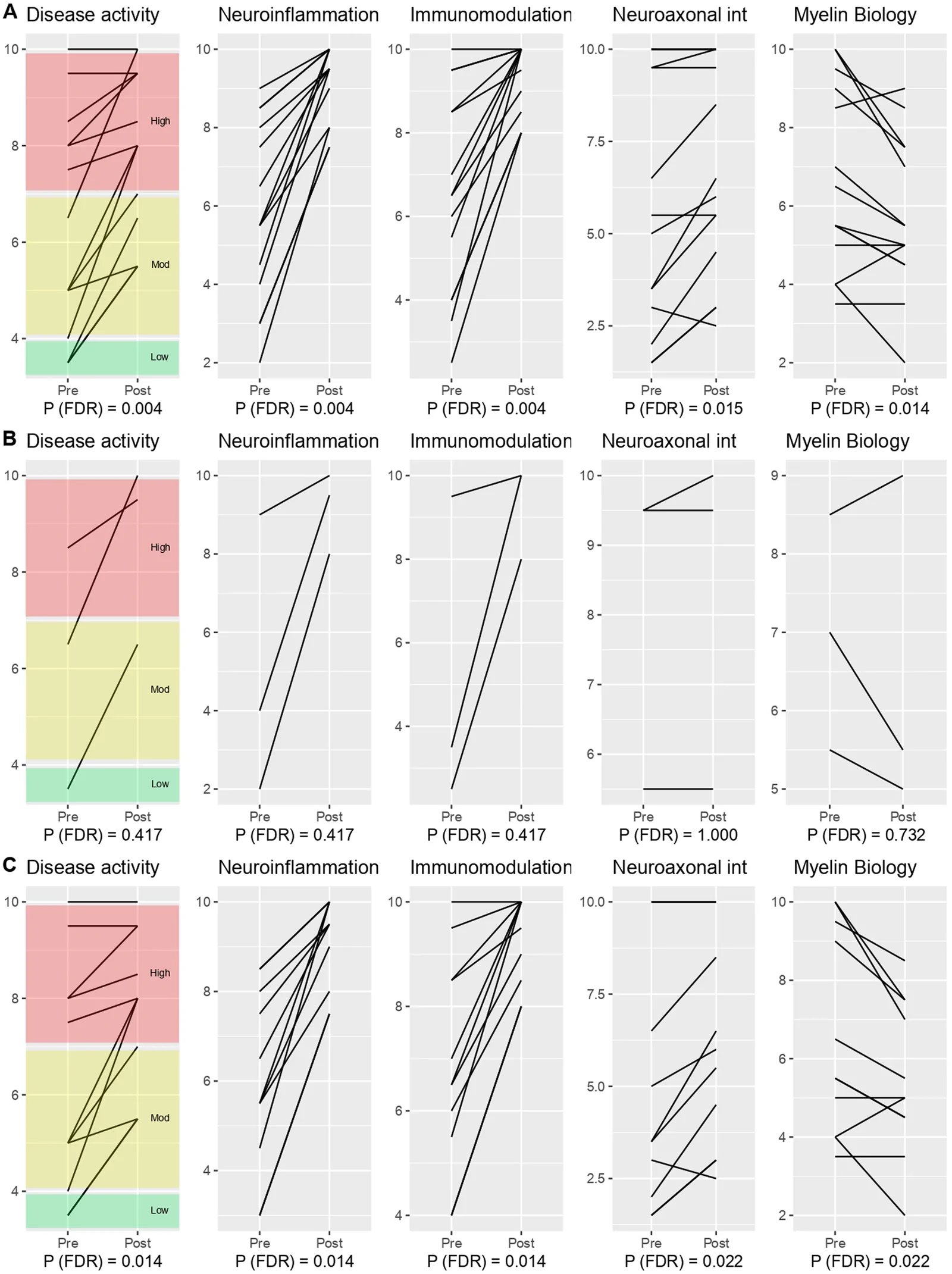
A 3-day course of high-dose IVMP causes significant change in Octave MSDA disease activity scores in (A) all patients with acute relapses of RMS; (B) patients with RMS without evidence of inflammatory activity on MRI; (C) patient with RMS with new gadolinium-enhancing lesions on MRI. Paired Wilcox rank-sum test was used to assess statistical significance and *p*-value for each test is provided below each graph. Disease Activity score levels (high, moderate, and low) are marked on each graph according to validated clinically meaningful categories.

The impact of IVMP on the composite MSDA scores (overall disease activity, neuroinflammation, immunomodulation, neuroaxonal integrity, and myelin biology) in the RMS subgroup was assessed. After IVMP, we observed statistically significant increases in overall disease activity score, neuroinflammation score, immunomodulation score, neuroaxonal integrity score. We observed a statistically significant decrease in Myelin biology score (Figure 2A). We wanted to focus on MSDA scores of patients without confirmed active Gad+ lesions on MRI and observed similar trends though without statistical significance in a limited three-patient cohort (Figure 2B). A group of 10 patients with Gad+ lesions on MRI exhibited patterns similar to the entire RMS group as well (Figure 2C).

**Figure 3.**
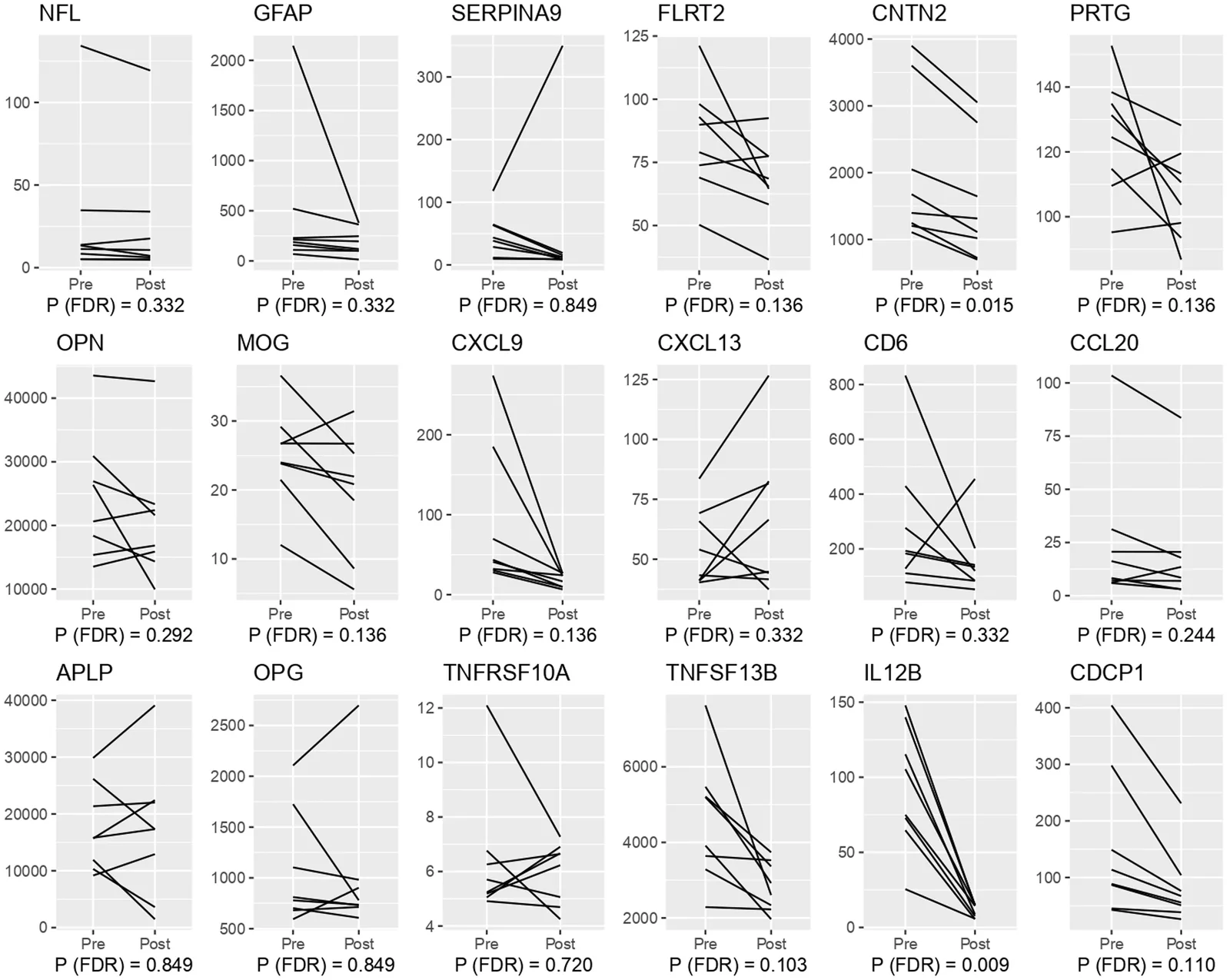
High-dose corticosteroids cause significant change in multiple serum biomarkers in patients with non-RMS patients. Absolute serum concentrations are presented. *P*-value is derived from paired *T*-test comparisons with FDR multiple comparisons correction.

To explore whether IVMP influences the same proteins in non-RMS demyelinating disease, we evaluated paired pre and post-IVMP concentrations in eight patients with MOGAD, NMOSD, idiopathic optic neuritis, and transverse myelitis. In this pooled non-RMS group, two serum proteins showed directionally similar responses to corticosteroids as in RMS, with significant decreases in Contactin-2 and Interleukin-12B (Figure 3).

## Discussion

In this pilot prospective study of hospitalized patients with acute relapses of primary demyelinating disease, we characterized short-term changes in a multi-protein serum biomarker panel in response to a standard 3-day course of high-dose IVMP.

In RMS, we found that high-dose IVMP induced rapid, statistically significant decreases in a set of proteins including GFAP, Serpin A9, FLRT2, contactin2, MOG, CXCL9, CD6, BAFF, IL-12B, and CDCP1. Some of these (e.g., BAFF, IL-12B, CXCL9, and CD6) are consistent with known glucocorticoid effects on lymphocyte trafficking, cytokine signaling, and B-cell survival.^3–11^ Others, particularly GFAP, FLRT2, contactin-2, and CDCP1, represent potentially novel corticosteroid-responsive markers in demyelinating disease. Down-regulation of these proteins over only three days in a group of patients with a variety of relapse severity and localization suggests they may be acutely sensitive to high-dose steroids, independent of longer-term disease activity. Further mechanistic studies are needed to examine the role of corticosteroid effects in astrocytic activation and integrity (GFAP), cellular adhesion (FLRT2 and contactin-2) and alternative CD6 receptor pathway activation (CDCP1).

Octave MSDA has been validated in a large sample, multicenter study and it's clinical utility has been recently published by several independent groups.^12,13^ Our project underscores an important consideration when interpreting this test in clinical practice. We observed a discordance between individual protein changes and composite MSDA scores. Despite decreases in several inflammatory and immune-related proteins, the MSDA disease activity, neuroinflammation, immunomodulation, and neuroaxonal integrity scores increased significantly after IVMP, while the myelin biology score decreased. We acknowledge that observed differences in MSDA disease activity scores between samples/groups should be interpreted in light of established test‒retest variability. In a recent independent reliability study of clinically stable RMS patients, the disease activity score showed high test‒retest reliability (*r* = 0.92) and a minimum detectable change (MDC95) of 1.24 units.^14^ In our cohort, the observed between-timepoint difference in composite scores exceeded this MDC95 threshold, suggesting that the observed difference likely reflects true biological change beyond measurement variability. This pattern strongly suggests that corticosteroid-driven shifts in specific analytes can perturb the composite scoring algorithm in ways that may not reflect true clinical or radiographic disease activity in the immediate post-treatment window. Particularly, the observed rapid reductions in BAFF (TNFSF13B), IL-12B, and CDCP1—biomarkers that inversely correlate with disease activity in the Octave MSDA panel—are likely contributing to the observed paradoxical effect. Clinicians should therefore be cautious in interpreting MSDA scores obtained shortly after high-dose corticosteroid therapy and consider retesting once steroid effects have waned.

The exploratory analyses in non-RMS demyelinating conditions indicated that two proteins (contactin-2 and IL-12B) exhibit corticosteroid-responsive decreases across MOGAD, NMOSD, and idiopathic optic neuritis/transverse myelitis. This cross-diagnostic responsiveness underscores that certain biomarkers may primarily reflect treatment effects rather than disease-specific pathobiology when measured in close temporal proximity to high-dose steroids. At the same time, it suggests potential shared immunopathological pathways modulated by corticosteroids across demyelinating phenotypes. It is important to note that the Octave MSDA panel used for biomarker analysis in our study, it's composite scores, and reference ranges have been developed and validated only in patients with multiple sclerosis. Further, large cohort studies examining serum biomarkers each unique disease state are needed to place our results in appropriate mechanistic context.

Our study has important limitations. The sample size is small, particularly for MOGAD and NMOSD, limiting power and precluding robust multivariable modeling or detailed phenotype stratification. Follow-up was restricted to the immediate post-IVMP period, so we cannot comment on the duration of steroid-induced biomarker changes or their association with clinical recovery or MRI outcomes. In our cohort, only eight patients got follow-up biomarker panel evaluation in an outpatient setting following their admission. Among those patients, only one MS patient and one ULON patient were not receiving ongoing immunotherapy during follow-up biomarker sampling. Further prospective studies are needed to evaluate dynamics of biomarker recovery following IVMP therapy. We also did not systematically evaluate the impact of prior or concurrent DMTs on biomarker responses.^15^ MRI evaluation of our patients was based on clinical localization, which may have led to underdiagnosis of new and/or enhancing lesions in our cohort. Additionally, variable MRI resolution and presence of artifacts may have affected detection of new lesions. Localization, volume of the affected nervous tissue, and chronicity of symptoms can all potentially affect multiple biomarkers. A larger cohort study is needed to evaluate effects of these factors as covariates in serum biomarker dynamics. Among MSDA composite scores, only disease activity score has been validated against a clinical metric in a prior study, therefore interpretation of other pathway scores remains limited. Finally, because we used a single commercial panel, our conclusions may not generalize to other biomarker platforms.

Despite these limitations, this pilot work has several practical implications. First, timing of serum biomarker sampling relative to high-dose corticosteroid administration is likely critical for both clinical and research use. Second, composite biomarker scores that integrate multiple analytes may be particularly vulnerable to transient treatment effects and should be interpreted in context. Third, the identification of contactin-2 as a consistently steroid-responsive protein across RMS and non-RMS conditions highlights potentially novel interactions between corticosteroid signaling and pathways related to cell adhesion and axoglial biology, meriting further mechanistic investigation.

## Conclusions

High-dose IV methylprednisolone induces rapid, measurable changes in multiple serum biomarker proteins and in composite MSDA scores in patients with acute relapses of RMS and other autoimmune demyelinating disorders. Some of these changes, particularly in GFAP, FLRT2, contactin-2, and CDCP1, have not been previously described in this context. Corticosteroid exposure should therefore be carefully considered when ordering and interpreting serum biomarker testing in the acute setting, and composite scores may be artificially elevated immediately after treatment due to steroid-dependent shifts in individual analytes. Larger, longitudinal studies are needed to validate these findings, define optimal sampling windows, and evaluate the diagnostic and prognostic utility of individual biomarkers in MOGAD, NMOSD, and other non-RMS demyelinating conditions.

## Supplemental Material

sj-docx-1-mso-10.1177_20552173261487037 - Supplemental material for Acute effects of high-dose corticosteroids on serum biomarker profiles in primary CNS demyelinating disease: A pilot study Supplemental material, sj-docx-1-mso-10.1177_20552173261487037 for Acute effects of high-dose corticosteroids on serum biomarker profiles in primary CNS demyelinating disease: A pilot study by Anastasia P Chumakova, Michael Demetriou and Michael Sy in Multiple Sclerosis Journal – Experimental, Translational and Clinical
