# Supplementary material for: Acute effects of high-dose corticosteroids on serum biomarker profiles in primary CNS demyelinating disease: A pilot study
Source: Mult Scler J Exp Transl Clin. 2026 Sep 7;12(3):20552173261487037. doi: 10.1177/20552173261487037 (PMC13554623; doi:10.1177/20552173261487037)
Supplement: Supplementary file 1 [file sj-docx-1-mso-10.1177_20552173261487037.docx]

**Supplemental figure 1**

High-dose corticosteroids cause significant change in multiple serum biomarkers in patients with RMS with evidence of inflammatory activity on MRI (N = 10). Absolute serum concentrations are presented. P-value is derived from paired T-test comparisons with FDR multiple comparisons correction.
